# Adverse drug events with hyperkalaemia during inpatient stays: evaluation of an automated method for retrospective detection in hospital databases

**DOI:** 10.1186/1472-6947-14-83

**Published:** 2014-09-12

**Authors:** Grégoire Ficheur, Emmanuel Chazard, Jean-Baptiste Beuscart, Béatrice Merlin, Michel Luyckx, Régis Beuscart

**Affiliations:** 1 Department of Public Health, EA 2694, University of Lille Nord de France, F-59000 Lille, France; 2 Pharmacology Laboratory, Denain General Hospital, F-59220 Denain, France

## Abstract

**Background:**

Adverse drug reactions and adverse drug events (ADEs) are major public health issues. Many different prospective tools for the automated detection of ADEs in hospital databases have been developed and evaluated. The objective of the present study was to evaluate an automated method for the retrospective detection of ADEs with hyperkalaemia during inpatient stays.

**Methods:**

We used a set of complex detection rules to take account of the patient’s clinical and biological context and the chronological relationship between the causes and the expected outcome. The dataset consisted of 3,444 inpatient stays in a French general hospital. An automated review was performed for all data and the results were compared with those of an expert chart review. The complex detection rules’ analytical quality was evaluated for ADEs.

**Results:**

In terms of recall, 89.5% of ADEs with hyperkalaemia “with or without an abnormal symptom” were automatically identified (including all three serious ADEs). In terms of precision, 63.7% of the automatically identified ADEs with hyperkalaemia were true ADEs.

**Conclusions:**

The use of context-sensitive rules appears to improve the automated detection of ADEs with hyperkalaemia. This type of tool may have an important role in pharmacoepidemiology via the routine analysis of large inter-hospital databases.

## Background

Adverse drug reactions (ADRs) and adverse drug events (ADEs) are involved in nearly 100,000 deaths in the United States of America each year, [1,2] and may have accounted for 5% of inpatient deaths [3] in a Swedish hospital. In France, a nationwide survey revealed that the incidence of severe ADEs during hospitalization is 7.6 per 1000 inpatient days [4]; of these, three severe ADEs per 1000 inpatient days may be preventable. Accordingly, ADEs constitute a major public health issue.

### The definition of ADRs

The World Health Organisation (WHO) defines ADRs as “a response to a medicinal product which is noxious and unintended, and which occurs at doses used in man for prophylaxis, diagnosis or therapy” [5]. This definition refers to reactions that occurs at “normal” therapeutic dose levels and thus excludes medication errors.

### The definition of ADEs

An ADE can be defined as “an injury resulting from medical intervention related to a drug” (in contrast to an injury resulting from “the underlying condition of the patient”) [6]. Thus, ADEs include both ADRs and prescription errors (overdoses). Knowing the proportion of ADEs related to medication errors is essential from an epidemiological point of view because the latter can (at least in theory) be avoided. For this reason, we chose to study ADEs in general (i.e. events including preventable ADEs and ADRs).

According to the United States Food and Drug Administration, an adverse event is considered to be serious when the outcome is “death”, a “life-threatening” situation, “hospitalization (initial or prolonged)”, “disability or permanent damage”, “congenital anomaly/birth defect” or “intervention to prevent permanent impairment or damage” [7].

### Case of ADEs with hyperkalaemia

Drug-induced hyperkalaemia is a significant issue [8]. The main complications of hyperkalaemia are cardiac diseases (such as conduction disorders, ventricular fibrillation, and cardiac arrest). Electrocardiographic features of hyperkalaemia include a sharp and broad T-wave, QRS widening and disappearance of the T-wave.

### Detection of ADEs

#### Retrospective detection of ADEs

In post-marketing (phase IV) studies, pharmacovigilance data are typically obtained from spontaneous reports by individual health professionals faced with an anomaly that they consider to be an ADR. These reports are not exhaustive and are known to suffer from underreporting bias [9,10]. One can therefore reasonably consider that conventional pharmacovigilance data (i) does not encompass all ADRs and (ii) provides little information on ADEs. Moreover, these declarative data have already been interpreted and do not contain any control cases. These limitations increase the potential value of gathering objective data, such as electronic medical records from hospital information systems. In general, the data in these systems (drug administrations, laboratory results and administrative data) have been routinely and exhaustively collected and are very appropriate for retrospective cohort studies using alternative signal discovery approaches, such as data mining [11-15]. Electronic medical records constitute a major resource for observational, post-marketing analyses.

Given that ADEs are rare, it is necessary to enhance the power of statistical analyses by building inter-hospital databases. The European Union’s PSIP research project [16] (which encompasses this study) has enabled the construction of this type of database. Furthermore, the PSIP project has prompted the development of a custom common data model [17], a method for solving the semantic interoperability issues that affect laboratory results databases [18], and a method for free-text automated de-identification [19].

#### Computerized tools for ADE detection

A number of prospective tools for detecting ADEs have been developed and evaluated [20]. In most cases, these tools combined computerized physician order entries with a clinical decision support system that includes a set of detection rules. The tools provide real-time alerts for modifying prescriptions and preventing ADEs. These prospective tools are based on detection rules that can be used to prevent ADE occurrence or even help the physician to respond appropriately if an ADE occurs. The tools’ overall level of performance depends on the quality of the implemented detection rules. Each detection rule is composed of a set of causes leading to an outcome (equation 1):

(1) $$\mathrm{Caus}e_{1}\cap \ldots \cap \mathrm{Caus}e_{n}\to \mathrm{Outcome}$$

### Evaluation of ADE detection systems

#### Metric for evaluating rules

Rules are usually evaluated in terms of their level of precision (i.e. their predictive positive value, PPV) [21]. This reflects the degree of confidence that can be attributed to the rule (i.e. the proportion of true ADEs among those automatically detected). In a screening procedure, the results in terms of precision must always be considered with those concerning the recall (i.e. sensitivity). This reflects the system’s capacity to detect the ADEs that have occurred). Recall and precision are defined in the Methods section. Recall is difficult to evaluate because ADEs are rare events; for this reason, a large review of inpatient stays is required. In the most extreme case, a rule that could detect ADEs in every inpatient stay would have a recall of 100% and a precision close to 0%. Conversely, a rule that could only detect very obvious ADE cases would have a recall of 100% but a recall close to 0%. Hence, a tool’s overall quality is always a compromise between these two parameters, with the exact balance depending on the tool’s future use.

In studies dealing with automated detection of ADEs with hyperkalaemia, recall and precision have not always been assessed. Although Dormann [22] computed both recall and precision, Brown [23] and Raschke [24] only computed the recall. A series of empirical assessments [25] revealed that high false-positive rates (i.e. a low PPV, with high noise levels) still constitute a major limitation of these detection methods.

### The nature of detection rules

In the field of prospective detection, Schedlbauer *et al*. [26] (adapted from Kuperman *et al*. [27]) distinguished between two type of drug alert. Firstly, “basic drug alerts” are rules that involve (amongst others) drug-drug interactions [28-31] or drug allergy warnings [29]. Secondly, “advanced drug alerts” involve (amongst others) drug-lab alerts [29,31,32], drug age alerts [29] and dosing guidelines [28,29,32].

The prescription of an antidote can also be used as a trigger for ADE detection [31]. This kind of method was not presented in Schedlbauer’s review because the latter only covered the prospective detection of ADEs.

It is noteworthy that only drug-lab alerts describe both a cause (drug prescription) and a potential outcome (an abnormal laboratory test). However, there is no information on the chronological link between validation of the cause and occurrence of the potentially abnormal laboratory test (since the order and the time interval are not specified).

Furthermore, experts performing a chart review are always required to assess the putative causal relationship between a drug and an ADEs in a complex context that combines clinical data (mainly diagnoses and symptoms) and laboratory results. We hypothesized that such contexts could favour the occurrence of ADEs.

In order to take account of the chronology of events and the clinical and biological context, we have developed a set of complex detection rules (described in detail below). With a view to linking these rules to the types of alert proposed by Schedlbauer [26], we included (i) basic or advanced drug alerts, (ii) items related to the clinical and biological context and (iii) a check on the chronology of the events (particularly the time interval between the drug administration and the ADE.

### Assessment of drug causality

There are many methods for assessing drug causality, and the degree of agreement between these methods varies significantly. The three main methods for validating individual cases [33] are probabilistic approaches, expert opinion and algorithmic-based approaches. The probabilistic approach is the most reproducible method, although it is not used routinely because it involves a complex modelisation. In contrast, expert opinion is subjective and thus poorly standardized. Hence, standardized, algorithm-based approaches are most commonly used. These are based on questionnaires and estimate the likelihood of whether an ADR has occurred. For example, Naranjo [34], Kramer [35] and Bégaud [36] have built algorithms originally to confirm potential ADRs. It is noteworthy that Kramer’s algorithm (used in the present study) specifies that the abnormal clinical manifestation (affecting the patient) is an abnormal symptom (or diagnosis) and/or an abnormal laboratory test.

In the literature, sets of detection rules vary greatly in terms of their performance levels. However, a set of rules’ PPV appears to be closely correlated with the method implemented for drug causality assessment. This heterogeneity has been assessed by Handler *et al*. [21] and was confirmed in the case of rules for hyperkalaemia in particular.

### Objective

The objective of the present study was to evaluate an automated method for the retrospective detection of ADEs in inpatient stays, with a focus on ADEs with hyperkalaemia. The method applied a set of complex detection rules to a hospital database. The quality of the set of detection rules was evaluated by comparison with an expert review (based on Kramer’s algorithm). The ADEs were classified as serious or non-serious.

## Methods

### Inpatient stays used for the study

The dataset comprised inpatient stays (defined here as at least two consecutive overnight hospitalizations) in a French general hospital during the first nine months of 2010. All the data were obtained from the electronic medical records stored in the hospital’s health information system (sourced in the departments of geriatrics, internal medicine, pulmonology, cardiology, gastroenterology and surgery). The number of inpatient stays (with a length of stay greater than two days) in the database over this period is 3,444. The de-identification of the medical records was performed using FASDIM algorithm [19]. The ethical agreement to analyze the medical records was given by the French Commission Nationale Informatique et Libertés.

### Rule building and analysis

The different types of hospital data are presented in Table 1, together with examples of rules conditions those data enable to build.

**Table 1:** Types of data contained in the electronic medical records

**Type of data**	**Terminology**	**Chronological relationship with other events**	**Example of condition**
Drug prescription	ATC [37]	Available	Potassium-sparing diuretic = 1
Laboratory result	C-NPU [38]	Available	Cytolytic hepatitis = 1 (i.e. transaminases > three times the upper normal limit)
Diagnosis	ICD-10 [39]	Not available	Urinary infection = 1
Demographic characteristic	Local terminology	Available	Age >70

The detection rules were based on aggregated variables, i.e. groups of codes chosen by a committee of experts. For example, the variable “potassium-sparing diuretic” refers to all ATC codes compatible with this kind of drug. Similarly, the variable “cytolytic hepatitis” refers to all abnormal laboratory tests (using the C-NPU terminology) that are compatible with this syndrome (alanine transaminase or aspartate transaminase levels greater than three times the upper normal limit), and “urinary infection” includes all ICD-10 codes compatible with this diagnosis.

### Properties of rules used for the automated review

In the present study, a committee of expert pharmacologists and pharmacists used the aggregated variables to build a set of complex detection rules.

The most important properties of the complex detection rules are as follows:

• The rules’ “cause conditions” included (i) a drug known to be associated with a risk of hyperkalaemia and (ii) a context variable that favours the occurrence of hyperkalaemia. Drugs with a risk of hyperkalaemia included renin angiotensin system inhibitors, beta blockers, potassium-sparing diuretics, potassium chloride, non-steroidal anti-inflammatory drugs and high-molecular-weight heparin. As mentioned above, these drugs or drug classes were built by aggregation of ATC codes. The three context variables favouring the occurrence of hyperkalaemia were diabetes (identified with ICD-10 codes), kidney failure (including functional kidney failure and the latter’s causes, such as mitral insufficiency and congestive heart failure, as identified with ICD-10 codes and laboratory results) and age > 70 (derived from demographic characteristics).

• The rule’s outcome is always hyperkalaemia (defined as a plasma potassium level greater than 5.3 mmol/l).

• The chronology of the rule’s variables: the “cause conditions” comprise several causes and are met when all the subconditions are met. Furthermore, all the subconditions must be met in the five days prior to occurrence of the outcome (hyperkalaemia, in this case). This time interval seems to be appropriate for taking account of the drugs with the longest half-life (some renin angiotensin system inhibitors and some potassium-sparing diuretics).

An example of a set of complex detection rule is presented in Equation 2. The set includes 18 rules (specified in Additional file 1) with the same outcome (hyperkalaemia).

(2) $$\begin{array}{c} \underset{\mathrm{Cause}\;\mathrm{conditions}}{\;\underset{︸}{\underset{\mathrm{context}\;\mathrm{condition}}{\underset{︸}{\mathrm{Renal}\;\mathrm{failure}}}\mathrm{AND}\;\underset{\mathrm{drug}\;\mathrm{prescription}}{\underset{︸}{\mathrm{Potassium}\;\mathrm{chloride}}}}}\;\underset{1-5\;\mathrm{days}\;\mathrm{after}}{\underset{︸}{\vec{\mathrm{AND}\;\mathrm{AFTER}}}}\underset{\mathrm{Outcome}=\mathrm{Expected}\;\mathrm{anomaly}}{\underset{︸}{\underset{\mathrm{abnormal}\;\mathrm{lab}\;\mathrm{test}}{\underset{︸}{\mathrm{Hyperkalemia}}}}} \end{array}$$

An inpatient stay was considered to be positive for a “complex detection rule” when all three conditions (“cause conditions” AND “outcome” AND “consistent chronology”) were met. It should be borne in mind that these rules were only applied retrospectively, since an outcome is always present. The presence of “cause conditions” means that a drug and a context favouring the hyperkalaemia must both be present in the five days prior to the outcome. After the set of rules had been elaborated by the expert pharmacists and pharmacologists, it was optimized by testing against a dataset of previous inpatient stays.

### Review of inpatient stays

An expert chart review and an automated review have been conducted, each one “blindly” on the other (i.e. each review was conducted while ignoring the results from the other review). The blind review in question is not about automated rule execution, which is obvious; instead, this blind review is about the preliminary stage of rule construction.

#### Automated detection of ADEs in inpatient stays

A set of scripts programmed in R [40] was used to scan the hospital database with the set of detection rules. The scripts work automatically with data that comply with the common model developed in the PSIP project and generate XML files as their output. A web-based “ADE Scorecards” tool [41] enables the expert to review the automatically detected cases and the corresponding, full electronic medical record.

#### Expert chart review of inpatient stays

The review was carried out by an expert physician and performed blindly from the results obtained by an automated analysis of inpatient stays. Kramer’s algorithm was used to assess drug causality [35]. For each reviewed stay, the expert had to answer one main question and two conditional questions:

• According to Kramer’s algorithm, does the inpatient stay correspond to either a “definite” ADE (Kramer score = +7 or +6) OR a “probable” ADE (Kramer score = +5 or +4)? YES / NO

• If YES:

•Which drug or drugs are responsible for this hyperkalaemia?

•Is hyperkalaemia (as an abnormal laboratory result) associated with an “abnormal symptom” for the patient?

### Contingency table with the results of the two reviews: computation of quality criteria for automated detection

In the present study, we present a contingency table to compare a diagnostic test featuring a binary answer (i.e. the automated detection of an ADE or not) with a reference answer (i.e. the expert review). The contingency table’s format is presented in Table 2. It is important to note that true positives are stays that are not only correctly identified as having an ADE but also feature the drug considered by the expert to have caused hyperkalaemia.

**Table 2:** The contingency table

	**ADE (hyperkalaemia), expert review**	**No ADE, expert review**
ADE (hyperkalaemia), automated detection	true positive (TP)	false positive (FP)
No ADE, automated detection	false negative (FN)	true negative (TN)

On the basis of the contingency table, equations 3 and 4 were respectively used to compute the recall and precision.

(3) $$\mathrm{Recall}=\frac{\mathrm{TP}}{\mathrm{TP}+\mathrm{FN}}$$

(4) $$\mathrm{Precision}=\frac{\mathrm{TP}}{\mathrm{TP}+\mathrm{FN}}$$

The set of complex detection rules” is used to detect cases of ADEs. The *a priori* system favours recall over precision so as not to miss an ADE. In this context, the harmonic mean (the F-measure) is not a relevant criterion. It is important to note that (i) any stay that triggers a rule has at least one hyperkalaemia event (since the latter is the rule’s outcome) and (ii) any stay selected by the expert also necessarily features at least one hyperkalaemia event (since it is the focus of the present study).

Thus, the contingency table can be completed by reviewing only inpatient stays that feature a hyperkalaemia event. This was undertaken in the analysis - especially for the expert review. It should be noted that the ability to detect hyperkalaemia *per se* does not generally need to be assessed; it is an objective element obtained by simply querying the laboratory results database. In contrast, it is essential to assess the automated system’s ability to highlight hyperkalaemia caused by a drug that is specified by at least one rule.

Lastly, as mentioned above, the expert is asked to specify whether each ADE was serious or not.

## Results

The expert chart review focused on all stays presenting hyperkalemia, i.e. a total of 120 inpatient stays. On average, the expert took 15 minutes to review of each impatient stay. In comparison, the automated detection system took a few minutes to “review” (i.e. process) the entire database of 3444 stays.

### Expert review

The expert review highlighted 57 ADEs with hyperkalaemia. Table 3 presents the characteristics of patients with an ADE and those without. A high proportion of patients with an ADE had acute kidney failure (44%). Similarly, a high proportion had heart failure (26%). This is probably due to functional kidney failure resulting from heart failure.

**Table 3:** Characteristics of patients with or without an ADE, based on the expert review

	**ADE (hyperkalaemia in the presence or absence of an abnormal symptom) in the expert review n1 = 57**	**No ADE (with hyperkalaemia) in the expert review n2 = 3387**
Age (years)	74.6 ± 2.4	67.4 ± 0.3
Men	26%	42%
Acute kidney failure	44%	11%
Muscle damage	2%	2%
Diabetes	21%	11%
Heart failure	26%	6%
Number of drugs administered	7.7 ± 0.5 (median 7.5)	4.9 ± 0.1 (median 4.7)
Length of stay (days)	14.6 ± 1.3 (median 12)	9.7 ± 0.1 (median 8)

The results obtained for all ADEs are shown in Table 4. The automated detection system flagged up 80 ADEs with hyperkalaemia, including 51 of the 57 identified by expert review (yielding a recall of 89.5%). Of the 80 automatically identified ADEs, 51 had the correct drug allocation (yielding a precision of 63.7%).

**Table 4:** Evaluation of automated detection of ADEs with hyperkalaemia

	**ADE (with hyperkalaemia), expert review**	**No ADE (with hyperkalaemia), expert review**	**Total**
ADE (with hyperkalaemia), automated detection	51	29	80
No ADE (with hyperkalaemia), automated detection	6	3358	3364
Total	57	3387	3444

Table 5 compares the automated detection and the expert review for each hospital department.

**Table 5:** Comparison of automated detection and expert review of data from each hospital department

**Hospital department**	**Stays**	**Stays with hyperkalaemia (HK)**	**ADE (with HK), expert review**	**ADE (with HK) automated detection**	**ADE (with HK), expert review and automated detection**
Geriatrics	257	12 (4.6%)	6 (2.3%)	9 (3.5%)	5 (1.9%)
Gastroenterology and Cardiology	970	46 (4.7%)	31 (3.1%)	31 (3.1%)	25 (2.5%)
Internal medicine	761	21 (2.7%)	10 (1.3%)	15 (1.9%)	10 (1.3%)
Pulmonology	655	24 (3.6%)	13 (1.9%)	19 (2.9%)	11 (1.6%)
Surgery	933	24 (2.5%)	2 (0.2%)	10 (1.0%)	2 (0.2%)
Total (individual stays)	3444	120 (3.4%)	57 (1.6%)	80 (2.3%)	51 (1.4%)

### Detection of serious ADEs

The expert chart review identified three serious ADEs (as defined in the introduction) with hyperkalaemia. All three cases were automatically identified with the correct drug allocation. The ADEs’ outcomes and the drugs involved are specified in Table 6. Although two of the three patients died, it must be borne in mind that both were suffering from serious illness.

**Table 6:** Characteristics of patients with ADEs with abnormal symptom

**Age**	**Gender**	**Length of hospital stay (days)**	**Kalaemia (mmol/l)**	**Cause of the ADE**	**Severe arrhythmia**	**Transfer to the intensive care unit**
50.3	M	7	8.0	Angiotensin receptor blocker (sartan) and potassium chloride per os	Yes	No
96.8	F	5	6.1	ACE inhibitor	Yes	No
70.6	M	24	7.7	Intravenous potassium chloride	Yes	No

## Discussion

In the present study, we used a set of complex detection rules to retrospectively detect ADEs with hyperkalaemia in a hospital database. This automated detection step yielded high recall and precision values, relative to expert chart review. In terms of the recall, 89.5% of ADEs with hyperkalaemia (regardless of the presence or absence of an abnormal symptom). As a result, all the serious ADEs were automatically detected: this is an important result, even though the small number of cases prevents us from extrapolating this finding to other situations. In terms of precision, 63.7% of the automatically identified ADEs with hyperkalaemia were true ADEs.

Our present results appear to confirm the need to take account of a patient’s clinical and biological context when seeking to automatically detect particular ADEs. Patients with normal renal function are unlikely to present hyperkalaemia, since excess potassium is rapidly eliminated by the kidneys. Conversely, kidney failure is rarely the sole cause of hyperkalaemia (except in cases of end-stage renal disease). Hence, it is reasonable to expect hyperkalaemia to occur when a drug favouring hyperkalaemia is prescribed in a context of kidney failure. Kidney failure (and particularly acute kidney failure) therefore appears to be a necessary (but not sufficient) condition for the occurrence of hyperkalaemia.

The present work also illustrates the role of laboratory results in the automated detection of ADEs. Laboratory results were used both as conditions and outcomes of our set of complex detection rules; these results appear to be relevant indicators for detecting ADEs [42] in general and hyperkalaemia (itself a complication) in particular. Moreover, cardiac symptoms often occur after an abnormal laboratory test result has been observed, meaning that the latter approach may be more sensitive. Our finding is in agreement with the review by Handler et al. [21], which identified 36 unique ADE signals (10 medication levels, 19 laboratory values and 7 antidotes). Laboratory results are structured data and are available during inpatient stays. This is not the case for structured diagnostic information (e.g. diagnostic codes), which are usually generated after the stay and do not provide precise information at the time of an ADE.

The rules built in the present study did not take antidotes into account. This was because we chose to build our rules were built to deal in both prospective [43] and retrospective ways. In another context, the incorporation of this type of outcome (such as the prescription of an antidote, e.g. a potassium chelator) could be useful.

Our present results can be compared with those of three similar studies that computed quality criteria for sets of detection rules. Firstly, Dormann [22] evaluated a computer-assisted monitoring system for the detection of ADRs in gastroenterology. The rules adopted were laboratory alerts and an ADR was defined “if the physician’s chart noted a change in drug regimen, additional laboratory tests or other diagnostic actions, subsequent and related to a specific ADR”. Two automated detection methods were employed. Method 1 had a precision of 36% for dyskalaemia and a recall of 91% for all ADRs. The corresponding values for Method 2 were 67% and for the precision (dyskalemia) and 40% for the recall (all ADRs). Secondly, Brown [23] presented the results of the Recognizing, Assessing and Documenting Adverse Rx events (RADARx) project. Again, the rules were laboratory alerts (for the “potassium” trigger) and the ADEs were validated according to Naranjo’s algorithm. Brown reported a precision of 11.1% (for potassium) but did not calculate the recall. Thirdly, Raschke [24] presented a “Computer Alert System to Prevent Injury From Adverse Drug Events”, with a rule for detecting “hyperkalaemia AND multiple drugs” (angiotensin-converting enzyme inhibitor, potassium chloride, potassium-sparing diuretics, trimethoprim sulphate or heparin sodium). Raschke’s assessment focused on the ability to prospectively identify a risky situation, rather than the ability to detect a prior ADE. Of the 69 alerts of this type, 41 alerts were true positive and 10 constituted a potential risk for the patient (that the physician had not recognized) but not a proven ADE. It is therefore difficult to compare Raschke’s results with our present results. The Precision found by authors is 59%, and Recall was not calculated. Raschke’s rules took account of hyperkalaemia and drug prescription but not the clinical and biological context of hyperkalaemia.

Consequently, our present results appear to be more satisfactory that those reported in the three above-mentioned studies. However, we cannot be sure that the same quality of results would be obtained with a set of detection rules for another outcome (i.e. another type of ADE). Furthermore, the low number of ADEs in this study means that it is difficult to generalize these results: ADEs are rare and so the construction of inter-hospital databases may provide more robust results.

These retrospective, complex detection rules might be of value in two situations. Firstly, expert chart review is a time-consuming task. Automated detection might enable a physician or pharmacologist to perform a more focused review of a few automatically detected stays. This approach would reduce the number of inpatient stays to be reviewed. This step can be envisaged because of our technique’s high recall. The tool presented here could also be used to generate retrospective alerts during hospitalization. A pharmacovigilance specialist could then review the alert and, if a drug is suspected to be responsible for an ADE, contact the patient’s attending physician [44]. Secondly, the performance of our set of complex detection rules shows that automated techniques could be used to estimate the occurrence of ADEs. If a technique’s precision and recall are constant, then the number of ADEs could be estimated as “n_automated detections_ × ∗ (Precision/Recall)”. This type of computerized tool may therefore have a role in hospital pharmacoepidemiology through the routine analysis of large, inter-hospital databases.

## Conclusions

The objective of the present study was to create and then evaluate complex detection rules that take account of the clinical and biological context in which a drug is prescribed and the chronological relationship between the cause and the expected outcome. The complex detection rules were evaluated against a nine-month database set of inpatient stays at a French general hospital. The method’s estimated recall and precision appear to be clinically relevant. A system based on these rules is now in routine use by the general hospital’s physicians and pharmacists and is being deployed in other hospitals in the region. We consider that this type of automated tool could have a role in increasing patient safety and quality of care.

## Competing interests

The authors declare that they have no competing interests.

## Authors’ contributions

GF suggested the use of complex detection rules to detect ADEs, carried out the design of the study, performed the statistical analysis and drafted the manuscript. EC participated in the data management (including the data model) and participated in the statistical analysis. BM built the rules and selected the method for assessing drug causality. JBB conducted the expert review and validated ADEs. ML helped to validate the rules. RB led the European PSIP project and helped to draft the manuscript. All authors have read and approved the final manuscript.

## Pre-publication history

The pre-publication history for this paper can be accessed here:

http://www.biomedcentral.com/1472-6947/14/83/prepub

## Supplementary Material

Additional file 1 **List of complex detection rules.** This file contains the 18 rules evaluated in this work. Click here for file
