# Supplementary material for: Adverse drug events with hyperkalaemia during inpatient stays: evaluation of an automated method for retrospective detection in hospital databases
Source: BMC Med Inform Decis Mak. 2014 Sep 12;14:83. doi: 10.1186/1472-6947-14-83 (PMC4164763; doi:10.1186/1472-6947-14-83)
Supplement: Additional file 1 — List of complex detection rules. This file contains the 18 rules evaluated in this work. [file 1472-6947-14-83-S1.docx]

| Rule id | Rule description |
| --- | --- |
| 1 | $\underset{\boldsymbol{context condition}}{\underbrace{\boldsymbol{Renal failure}}}\boldsymbol{+}\underset{\boldsymbol{drug prescription}}{\underbrace{\boldsymbol{Potassium chloride}}} \vec{\boldsymbol{THEN}} \underset{\boldsymbol{Expected anomaly}}{\underbrace{\boldsymbol{Hyperkalemia}}}$ |
| 2 | $\underset{\boldsymbol{context condition}}{\underbrace{\boldsymbol{Renal failure}}}\boldsymbol{+}\underset{\boldsymbol{drug prescription}}{\underbrace{\boldsymbol{Renin angiotensin system inhibitor}}} \vec{\boldsymbol{THEN}} \underset{\boldsymbol{Expected anomaly}}{\underbrace{\boldsymbol{Hyperkalemia}}}$ |
| 3 | $\underset{\boldsymbol{context condition}}{\underbrace{\boldsymbol{Renal failure}}}\boldsymbol{+}\underset{\boldsymbol{drug prescription}}{\underbrace{\boldsymbol{Beta blocker}}} \vec{\boldsymbol{THEN}} \underset{\boldsymbol{Expected anomaly}}{\underbrace{\boldsymbol{Hyperkalemia}}}$ |
| 4 | $\underset{\boldsymbol{context condition}}{\underbrace{\boldsymbol{Renal failure}}}\boldsymbol{+}\underset{\boldsymbol{drug prescription}}{\underbrace{\boldsymbol{Potassium sparing diuretic}}} \vec{\boldsymbol{THEN}} \underset{\boldsymbol{Expected anomaly}}{\underbrace{\boldsymbol{Hype}\boldsymbol{rkalemia}}}$ |
| 5 | $\underset{\boldsymbol{context condition}}{\underbrace{\boldsymbol{Renal failure}}}\boldsymbol{+}\underset{\boldsymbol{drug prescription}}{\underbrace{\boldsymbol{Non-steroidal anti-inflammatory}}} \vec{\boldsymbol{THEN}} \underset{\boldsymbol{Expected anomaly}}{\underbrace{\boldsymbol{Hyperkalemia}}}$ |
| 6 | $\underset{\boldsymbol{context condition}}{\underbrace{\boldsymbol{Renal failure}}}\boldsymbol{+}\underset{\boldsymbol{drug prescription}}{\underbrace{\boldsymbol{High molecular weight heparin}}} \vec{\boldsymbol{THEN}} \underset{\boldsymbol{Expected anomaly}}{\underbrace{\boldsymbol{Hyperkalemia}}}$ |
| 7 | $\underset{\boldsymbol{conte}\boldsymbol{xt condition}}{\underbrace{\boldsymbol{Diabetes}}}\boldsymbol{+}\underset{\boldsymbol{drug prescription}}{\underbrace{\boldsymbol{Potassium chloride}}} \vec{\boldsymbol{THEN}} \underset{\boldsymbol{Expected anomaly}}{\underbrace{\boldsymbol{Hyperkalemia}}}$ |
| 8 | $\underset{\boldsymbol{context condition}}{\underbrace{\boldsymbol{Diabetes}}}\boldsymbol{+}\underset{\boldsymbol{drug prescription}}{\underbrace{\boldsymbol{Renin angiotensin system inhibitor}}} \vec{\boldsymbol{THEN}} \underset{\boldsymbol{Expected anomaly}}{\underbrace{\boldsymbol{Hyperkalemia}}}$ |
| 9 | $\underset{\boldsymbol{context condition}}{\underbrace{\boldsymbol{Diabetes}}}\boldsymbol{+}\underset{\boldsymbol{drug prescription}}{\underbrace{\boldsymbol{Beta blocker}}} \vec{\boldsymbol{THEN}} \underset{\boldsymbol{Expected anomaly}}{\underbrace{\boldsymbol{Hyperkalemia}}}$ |
| 10 | $\underset{\boldsymbol{context condition}}{\underbrace{\boldsymbol{Diabetes}}}\boldsymbol{+}\underset{\boldsymbol{drug prescription}}{\underbrace{\boldsymbol{Potassium sparing diuretic}}} \vec{\boldsymbol{THEN}} \underset{\boldsymbol{Expected anomal}\boldsymbol{y}}{\underbrace{\boldsymbol{Hyperkalemia}}}$ |
| 11 | $\underset{\boldsymbol{context condition}}{\underbrace{\boldsymbol{Diabetes}}}\boldsymbol{+}\underset{\boldsymbol{drug prescription}}{\underbrace{\boldsymbol{Non-steroidal anti-inflammatory}}} \vec{\boldsymbol{THEN}} \underset{\boldsymbol{Expected anomaly}}{\underbrace{\boldsymbol{Hyperkalemia}}}$ |
| 12 | $\underset{\boldsymbol{context condition}}{\underbrace{\boldsymbol{Diabetes}}}\boldsymbol{+}\underset{\boldsymbol{drug prescription}}{\underbrace{\boldsymbol{High molecular weight heparin}}} \vec{\boldsymbol{THEN}} \underset{\boldsymbol{Expected anomaly}}{\underbrace{\boldsymbol{Hyperkalemia}}}$ |
| 13 | $\underset{\boldsymbol{context condition}}{\underbrace{\boldsymbol{Age}>70}}\boldsymbol{+}\underset{\boldsymbol{drug prescription}}{\underbrace{\boldsymbol{Potassium c}\boldsymbol{hloride}}} \vec{\boldsymbol{THEN}} \underset{\boldsymbol{Expected anomaly}}{\underbrace{\boldsymbol{Hyperkalemia}}}$ |
| 14 | $\underset{\boldsymbol{context condition}}{\underbrace{\boldsymbol{Age}>70}}\boldsymbol{+}\underset{\boldsymbol{drug prescription}}{\underbrace{\boldsymbol{Renin angiotensin system inhibitor}}} \vec{\boldsymbol{THEN}} \underset{\boldsymbol{Expected anomaly}}{\underbrace{\boldsymbol{Hyperkalemia}}}$ |
| 15 | $\underset{\boldsymbol{context condition}}{\underbrace{\boldsymbol{Age}>70}}\boldsymbol{+}\underset{\boldsymbol{drug prescription}}{\underbrace{\boldsymbol{Beta blocker}}} \vec{\boldsymbol{THEN}} \underset{\boldsymbol{Expected anomaly}}{\underbrace{\boldsymbol{Hyperkalemia}}}$ |
| 16 | $\underset{\boldsymbol{context condition}}{\underbrace{\boldsymbol{Age}>70}}\boldsymbol{+}\underset{\boldsymbol{drug prescription}}{\underbrace{\boldsymbol{Potassium sparing diuretic}}} \vec{\boldsymbol{THEN}} \underset{\boldsymbol{Expected anomaly}}{\underbrace{\boldsymbol{Hyperkalemia}}}$ |
| 17 | $\underset{\boldsymbol{context condition}}{\underbrace{\boldsymbol{Age}>70}}\boldsymbol{+}\underset{\boldsymbol{drug prescription}}{\underbrace{\boldsymbol{Non-steroidal anti-inflammatory}}} \vec{\boldsymbol{THEN}} \underset{\boldsymbol{Expected anomaly}}{\underbrace{\boldsymbol{Hyperkalemia}}}$ |
| 18 | $\underset{\boldsymbol{context condition}}{\underbrace{\boldsymbol{Age}>70}}\boldsymbol{+}\underset{\boldsymbol{drug prescri}\boldsymbol{ption}}{\underbrace{\boldsymbol{High molecular weight heparin}}} \vec{\boldsymbol{THEN}} \underset{\boldsymbol{Expected anomaly}}{\underbrace{\boldsymbol{Hyperkalemia}}}$ |
